# Incidence, remission, and persistence of Raynaud’s phenomenon in the general population of northern Sweden: a prospective study

**DOI:** 10.1186/s41927-022-00272-0

**Published:** 2022-07-21

**Authors:** Albin Stjernbrandt, Hans Pettersson, Ronnie Lundström, Ingrid Liljelind, Tohr Nilsson, Jens Wahlström

**Affiliations:** Section of Sustainable Health, Department of Public Health and Clinical Medicine, Umeå University, 901 87 Umeå, Sweden

**Keywords:** Sweden, Longitudinal studies, Raynaud disease, Incidence, Remission, Spontaneous, Occupational exposure, Cold climate, Peripheral vascular diseases

## Abstract

**Background:**

Raynaud’s phenomenon is common condition, but little is known about the natural course. The primary aim of this study was to determine the incidence, remission, and persistence proportions of Raynaud’s phenomenon in the general population of northern Sweden. Secondary aims were to determine how individual and exposure factors affect the course of Raynaud’s phenomenon, and to assess gender differences.

**Methods:**

A prospective, survey-based, closed-cohort study was conducted on a sample of men and women between 18–70 years of age, living in northern Sweden. Data on Raynaud’s phenomenon characteristics and general health status were collected during the winters of 2015 (baseline) and 2021 (follow-up). Rates of incidence, remission, and persistence were calculated. Binary logistic regression was used to determine the association between baseline variables and the course of Raynaud’s phenomenon. Results: The study population consisted of 2703 women (53.9%) and 2314 men. There were 390 women (14.5%) and 290 men (12.7%) reporting Raynaud’s phenomenon in the follow-up survey. The annual incidence proportion was 0.7% among women and 0.9% among men (gender difference *p* = 0.04). The annual remission proportion was 4.4% and 5.5%, respectively (*p* = 0.05). Having sustained a cold injury affecting the hands since baseline was significantly associated with incident Raynaud’s phenomenon (OR 3.92; 95% CI 2.60–5.90), after adjusting for age and gender.

**Conclusions:**

In the general population of northern Sweden, Raynaud’s phenomenon is a common but variable condition, where symptoms may remit over time. Men had a higher incidence proportion than women. The results support a possible causal pathway where cold injury can precede the onset of Raynaud’s phenomenon.

**Supplementary Information:**

The online version contains supplementary material available at 10.1186/s41927-022-00272-0.

## Background

Raynaud’s phenomenon (RP) is a common condition, characterized by episodes of peripheral blanching of digits, which can be triggered by exposure to cold, hand-arm vibration, or emotional stress [1]. RP can be categorized into primary and secondary forms, where the former is a benign and idiopathic condition, whereas the latter is often more severe, progressive, and dependent on an underlying cause. Suffering from RP can affect both the work ability and quality of life [2]. The prevalence of RP has generally been reported to be higher among inhabitants in arctic and temperate climate compared to tropical climate, a consequence of cold exposure being the most common trigger for vasospastic attacks [1], but perhaps also an etiological factor [3]. The occurrence of RP in the Scandinavian general population has been reported to be about 12–14% [4, 5].

The natural course of RP is not well understood since population-based prospective studies are scarce. One such study conducted by Suter et al. in the U.S. followed 1358 subjects for a mean of seven years. They reported a cumulative incidence of 1.5–2.2% and a remission proportion of 64% [6]. In another similar study by Carpentier et al., conducted on 296 subjects living in a French alpine region, the cumulative incidence proportion was 3.5% and remission proportion 33% after 14 years of follow-up [7]. In addition, several smaller longitudinal clinical studies on RP subjects have been conducted, but mainly aimed at establishing a potential transition to secondary RP associated with rheumatic diseases [8–13]. However, few such studies have reported on any potential for remission [14–16]. No population-based longitudinal studies on RP have been conducted in a Scandinavian setting, which means that the course, and the influence of possible risk factors, has not been determined.

Regarding risk factors, numerous individual and external conditions have been reported to be associated with secondary RP [17, 18], among them rheumatic conditions, cardiovascular disease, migraines, tobacco use, cold exposure, and manifest cold injury [19–21]. Regarding the latter, mainly cross-sectional epidemiological studies have indicated a link between cold injury and RP. However, several authors have considered cold injury as an independent risk factor for contracting RP [3, 19, 22], while others have interpreted the results in the opposite direction, i.e. that manifest RP increases the risk of subsequent cold injury [23, 24]. To the authors’ knowledge, only one study has utilized longitudinal (retrospective) data, and reported an increased two-year cumulative incidence of extremity frostbite among those with vibration-induced secondary RP [25]. Thus, there is a need for population-based studies to determine the natural course of RP, and shed light on the time relation between cold injury and RP.

The primary aim of this study was to determine the incidence, remission, and persistence proportions of RP in the general population of northern Sweden. Secondary aims were to determine how individual and exposure factors affect the course of RP, and to assess gender differences.

## Methods

### Study design and setting

This prospective survey-based, closed-cohort study was part of the Cold and Health In Northern Sweden (CHINS) research project, which was initiated in 2015 to broadly explore adverse health effects from ambient cold exposure, and has previously been described in detail [4]. The study sample included men and women of working age at enrollment (18–70 years), living in northern Sweden, who were drawn from the national Swedish population register. Baseline data came from the first postal survey (CHINS2015), that was administered between February and May of 2015. Follow-up data was retrieved through a digital questionnaire (CHINS2021) that collected data between March and April of 2021. All subjects (N = 12,627) who had responded to the baseline questionnaire were invited by a postal query to respond to the follow-up questionnaire, with one postal reminder. Subjects were also given the option to respond to the questionnaire on paper, if reluctant or unable to answer digitally. The study protocol was approved by the Regional Ethical Review Board situated at Umeå University (DNR 2014–286-31 M) and the Swedish Ethical Review Authority (DNR 2020–06707).

### Variables and statistical analyses

Before the follow-up survey was administered, a statistical power analysis was performed, indicating that 400 responders with RP would be needed to reach a statistical power of 0.8 with an alpha of 0.05, assuming the same prevalence at baseline and follow-up. Social security number was used to merge data from both surveys. Since continuous variables were not normally distributed, data were described as median values and interquartile ranges (IQR), while categorical variables were presented as numbers and valid percentages (unless otherwise stated). Subjects with RP were defined by a positive response to a single questionnaire item that was present in both surveys: “Does one or more of your fingers turn white (as shown on picture) when exposed to moisture or cold?”, and this was supported by a standardized color chart, which has previously been shown to increase the accuracy in RP diagnosis [26]. Negating RP at baseline and reporting having the condition at follow-up was required for incident cases; remission was defined as reporting RP at baseline and negating the condition at follow-up. In the follow-up survey, study participants were also asked additional questions about year of first occurrence, demarcation, attack frequency, and distribution of RP. Occupational or leisure-time ambient cold exposure were assessed by two questionnaire items: “During work I am exposed to outdoor or cold environments” and “During leisure time I am exposed to outdoor or cold environments”. The answers were given on whole number numerical rating scales (NRS), ranging from one (“do not agree”) to ten (“fully agree”), and subsequently dichotomized based on the 50th percentile, but also analyzed continuously. Geographical location, as determined by postal code, was divided into three groups (rural alpine/inland/urban costal). Psychological stress was dichotomized so that “none/very little/some” was considered a negative response, and “quite a lot/very much” a positive response. Age was categorized into equal spans, body mass index (BMI) by clinically used thresholds for under- and overweight, but both also analyzed continuously. Additional binary baseline covariates included current daily smoking, current daily use of oral moist snuff (a common tobacco product in Sweden, typically placed between the upper lip and gum), hypertension, angina pectoris, myocardial infarction, stroke, diabetes mellitus, migraines, and cold injury affecting the hands. For the medical conditions, study participants were asked if they were treated with prescription drugs. Further, participants were asked to specify the severity of their cold injury (white spots/clear blisters/blood-filled blisters), and year of first occurrence. Finally, data from the follow-up survey was used to assess the presence of incident cold injury since baseline (negating injury at baseline and reporting injury at follow-up). Current occupation was specified in free-form text and manually coded in accordance with the International Standard Classification of Occupations (ISCO) [27]. The Mann–Whitney *U* test and Pearson’s chi square test (**χ**^2^) was used to determine gender differences for continuous and categorical variables, respectively. Binary logistic regression was used to determine the association between baseline variables and the course of RP, and presented as odds ratios (OR) with ninety-five percent confidence intervals (95% CI). A *p* value < 0.05 was considered statistically significant. Statistical analyses were performed using SPSS (version 27.0, IBM Corporation, Armonk, NY, USA).

## Results

### Recruitment

**Fig. 1:**
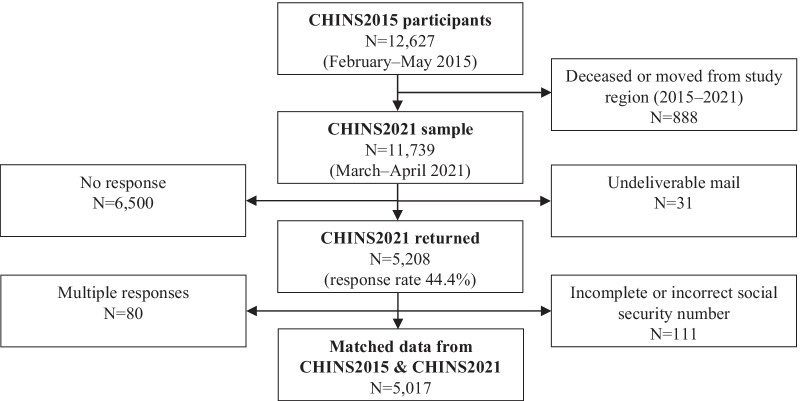
The data collection for the follow-up survey based on the participants of the baseline survey. *CHINS* cold and health in northern Sweden

There were 5208 responses to the follow-up survey (CHINS2021), yielding a response rate of 44.4% (Fig. 1). A detailed responder analysis is supplied in Additional file 1. Due to multiple responses and invalid social security numbers, 191 survey responses could not be matched to the original dataset, which left 5017 subjects available for analysis. Five subjects (0.01%) opted to respond on paper for the follow-up survey, while the rest answered to the digital questionnaire.

### Descriptive data

The final study population consisted of 2703 women (53.9%) and 2314 men. Other characteristics from the baseline survey are presented in Table 1. There were 390 women (14.5%) and 290 men (12.7%) reporting RP at follow-up. Detailed characteristics of RP from the follow-up survey are described in Table 2. Regarding longitudinal data, there were 396 women and 265 men with RP at baseline who also participated in the follow-up survey. Of these, 104 women and 88 men reported remission at follow-up. In addition, there were 96 women and 112 men who reported incident RP. Details on prevalence, incidence, remission, and persistence proportions for RP, categorized by gender, are shown in Table 3.

**Table 1:** Characteristics of the study participants in the baseline survey, separated by gender

Baseline variable	Women		Men	
	N (%)	Median (IQR)	N (%)	Median (IQR)
Study participants	2703 (53.9%)		2314 (46.1)	
Age (at enrollment)		53 (19)		56 (18)
*Anthropometry*				
Height (cm)		166 (8)		180 (9)
Weight (kg)		68 (17)		84 (17)
Body mass index (kg/m^2^)		24.7 (5.6)		26.1 (4.3)
*Region*				
Rural alpine	547 (20.2)		473 (20.4)	
Inland	691 (25.6)		600 (25.9)	
Urbanized coastal	1465 (54.2)		1241 (53.6)	
*Daily smoking*				
Never or former	2494 (93.0)		2190 (95.6)	
Current	188 (7.0)		101 (4.4)	
Cigarettes per day		10 (5)		10 (10)
*Snuff use*				
Never or former	2478 (92.7)		1825 (79.6)	
Current	196 (7.3)		469 (20.4)	
Snuff boxes per week		4 (3)		3 (3)
*Occupation (ISCO)*				
Armed forces	1 (0.1)		17 (0.8)	
Managers	132 (5.0)		127 (5.6)	
Professionals	751 (28.3)		342 (15.2)	
Technicians and associate professionals	255 (9.6)		339 (15.0)	
Clerical support workers	315 (11.9)		160 (7.1)	
Service and sales workers	455 (17.2)		158 (7.0)	
Skilled agricultural, forestry, and fishery workers	17 (0.6)		47 (2.1)	
Crafts and related trades workers	31 (1.2)		208 (9.2)	
Plant and machine operators and assemblers	38 (1.4)		252 (11.2)	
Elementary occupations	52 (2.0)		46 (2.0)	
Self–employed	42 (1.6)		58 (2.6)	
Sick leave	41 (1.5)		13 (0.6)	
Parental leave	22 (0.8)		0 (0)	
Students	98 (3.7)		56 (2.5)	
Retired	364 (13.7)		394 (17.5)	

*ISCO* international standard classification of occupations, *IQR* interquartile range

**Table 2:** Characteristics of Raynaud’s phenomenon in the follow-up survey, separated by gender

Variable	Women		Men		Gender difference ^a^
	N (%)	Median (IQR)	N (%)	Median (IQR)	*p* value
Age at onset (years)		31 (23)		35 (30)	< 0.01
Symptom duration (years)		21 (22.5)		23 (28)	< 0.01
*Year of latest vasospastic episode*					0.07
2021	244 (65.9)		184 (68.1)		
2020	84 (22.7)		46 (17.0)		
2019	12 (3.2)		13 (4.8)		
Earlier than 2019	30 (8.1)		27 (10.0)		
*Episode frequency compared to onset*					
Increased	93 (24.1)		103 (35.8)		< 0.01
Unchanged	124 (32.1)		89 (30.9)		
Decreased	131 (33.9)		73 (25.3)		
Not sure	38 (9.8)		23 (8.0)		
*Clear demarcation of paleness*					< 0.01
Yes	267 (69.2)		171 (60.0)		
No	29 (7.5)		58 (20.4)		
Not sure	90 (23.3)		56 (19.6)		
*Distribution of paleness right hand*					0.02
Only distal phalanges	155 (39.8)		123 (42.9)		
Distal and middle phalanges	181 (46.5)		117 (40.8)		
Distal, middle and proximal phalanges	28 (7.2)		24 (8.4)		
Only left-hand involvement	9 (2.3)		18 (6.3)		
Not sure	16 (4.1)		5 (1.6)		
*Distribution of paleness left hand*					0.16
Only distal phalanges	159 (41.0)		128 (45.2)		
Distal and middle phalanges	171 (44.1)		109 (38.5)		
Distal, middle and proximal phalanges	24 (6.2)		23 (8.1)		
Only right–hand involvement	18 (4.6)		18 (6.4)		
Not sure	16 (4.1)		5 (1.8)		
*Distribution compared to onset*					< 0.01
Increased	50 (13.0)		47 (12.3)		
Unchanged	199 (51.7)		133 (46.8)		
Decreased	63 (16.4)		69 (24.3)		
Not sure	73 (19.0)		35 (12.3)		

*IQR* interquartile range

^a^Mann–Whitney *U* test or Pearson’s χ^2^ test for women versus men

**Table 3:** Prevalence, incidence, remission, and persistence proportions for Raynaud’s phenomenon, separated by gender

Measure	Women ^a^	Men ^a^	Gender difference ^b^
	N (%)	N (%)	*p* value
Baseline RP	396/2703 (14.7)	265/2314 (11.5)	< 0.01
Incident RP ^c^	96/2307 (4.2)	112/2049 (5.5)	0.04
(per year) ^d^	16 (0.7)	19 (0.9)	
Remitted RP ^e^	104/396 (26.3)	88/265 (33.2)	0.05
(per year) ^d^	17 (4.4)	15 (5.5)	
Persistent RP ^f^	289/396 (73.0)	175/265 (66.0)	

*RP* Raynaud’s phenomenon

^a ^The figures are affected by item non-response, and absolute percentages are presented

^b ^Pearson’s χ^2^ test for women versus men

^c ^Number of incident cases divided by subjects at risk (total sample minus number of subjects with RP at baseline)

^d ^Cumulative estimate divided by six

^e ^Number of remitted cases (reporting RP at baseline and negating RP at follow-up), divided by the number of subjects with RP at baseline

^f ^Number of persistent cases (reporting RP at baseline and follow-up) divided by the number of subjects with RP at baseline

### Associated factors

There was a significant trend towards lower incidence of RP with increasing age (crude OR 0.99; 95% CI 0.98–0.99) (Table 4). After adjusting for age and gender, BMI was inversely associated with incident disease (adjusted OR 0.94; 95% CI 0.91–0.98), as was hypertension (adjusted OR 0.56; 95% CI 0.37–0.85). There were too few subjects reporting angina pectoris, myocardial infarction, and stroke to perform logistic regression (data not shown). Analyzing the course of established disease, age was associated with remitted RP (crude OR 1.02; 95% CI 1.01–1.04), as was BMI (adjusted OR 1.10; 95% CI 1.05–1.15) and hypertension (adjusted OR 1.88; 95% CI 1.25–2.83). Among subjects with hypertension, 976 (84.9%) indicated that they had been prescribed drug treatment, but detailed information regarding substance or dose were not collected.

**Table 4:** Binary logistic regression between independent variables at baseline and the course of Raynaud’s phenomenon

Baseline variable	OR for incident Raynaud’s phenomenon				OR for remitted Raynaud’s phenomenon			
	Incident	Never	OR (95% CI) ^a^	OR (95% CI) ^b^	Remitted	Persistent	OR (95% CI) ^a^	OR (95% CI) ^b^
*Age* (years)								
18–31	24	420	1.00	–	8	17	1.00	–
32–44	51	699	1.28 (0.77–2.11)	–	26	84	0.66 (0.26–1.70)	–
45–57	68	1282	0.93 (0.58–1.50)	–	53	182	0.62 (0.25–1.51)	–
58–70	65	1650	0.69 (0.43–1.11)	–	105	181	1.23 (0.51–2.95)	–
Continuous	–	–	0.99 (0.98–0.99)	–	–	–	1.02 (1.01–1.04)	–
*BMI* (kg/m^2^)								
< 20	13	139	1.44 (0.79–2.64)	1.52 (0.83–2.81)	4	31	0.35 (0.12–1.03)	0.40 (0.14–1.19)
20–25	101	1558	1.00	1.00	99	271	1.00	1.00
25–30	66	1617	0.63 (0.46–0.87)	0.62 (0.45–0.86)	62	133	1.28 (0.87–1.87)	1.18 (0.80–1.74)
> 30	26	679	0.59 (0.38–0.92)	0.60 (0.38–0.93)	25	26	2.63 (1.45–4.77)	2.57 (1.41–4.70)
Continuous	–	–	0.94 (0.91–0.98)	0.94 (0.91–0.98)	–	–	1.10 (1.06–1.16)	1.10 (1.05–1.15)
*Daily smoker*							445	
Never or former	197	3790	1.00	1.00	178	445	1.00	1.00
Current	11	241	0.88 (0.47–1.63)	1.00 (0.54–1.89)	13	17	1.91 (0.91–4.02)	1.87 (0.88–3.94)
*Daily snuff user*								
Never or former	170	3516	1.00	1.00	165	386	1.00	1.00
Current	37	514	1.49 (1.03–2.15)	1.35 (0.93–1.96)	25	76	0.77 (0.47–1.25)	0.72 (0.43–1.19)
*Hypertension*								
No	176	3064	1.00	1.00	130	377	1.00	1.00
Yes	28	945	0.52 (0.34–0.77)	0.56 (0.37–0.85)	61	84	2.11 (1.43–3.10)	1.88 (1.25–2.83)
*Diabetes mellitus*								
No	199	3841	1.00	1.00	186	453	1.00	1.00
Yes	7	185	0.73 (0.34–1.57)	0.80 (0.37–1.73)	3	9	0.81 (0.22–3.03)	0.73 (0.19–2.76)
*Migraines*								
No	188	3675	1.00	1.00	165	400	1.00	1.00
Yes	16	324	0.97 (0.57–1.63)	1.00 (0.59–1.71)	20	56	0.87 (0.50–1.49)	0.95 (0.54–1.64)
*Psychological stress*								
Low	171	3208	1.00	1.00	143	360	1.00	1.00
High	37	816	0.85 (0.59–1.22)	0.85 (0.59–1.23)	48	101	1.20 (0.81–1.78)	1.29 (0.86–1.92)
*Cold injury hands*								
No	169	3781	1.00	1.00	127	322	1.00	1.00
Yes	36	257	3.13 (2.14–4.59)	2.90 (1.97–4.27)	64	139	1.17 (0.81–1.67)	1.29 (0.88–1.89)
*Work cold exposure*								
NRS 1	112	2348	1.00	1.00	98	254	1.00	1.00
NRS 2–10 ^c^	87	1573	1.16 (0.87–1.55)	1.05 (0.78–1.42)	88	190	1.20 (0.85–1.69)	1.18 (0.82–1.69)
Continuous	–	–	1.04 (0.99–1.09)	1.02 (0.97–1.07)	–	–	1.05 (0.99–1.11)	1.05 (0.99–1.11)
Continuous working ^d^	–	–	1.03 (0.97–1.08)	1.01 (0.96–1.07)	–	–	1.08 (1.01–1.15)	1.07 (1.01–1.14)
*Leisure cold exposure*								
NRS 1–5	89	2039	1.00	1.00	75	199	1.00	1.00
NRS 6–10 ^c^	115	1962	1.34 (1.01–1.78)	1.28 (0.97–1.71)	114	257	1.18 (0.83–1.66)	1.20 (0.85–1.70)
Continuous	–	–	1.07 (1.01–1.13)	1.06 (1.01–1.12)	–	–	0.99 (0.93–1.06)	1.00 (0.93–1.07)

*BMI* body mass index, *NRS* numerical rating scale, *OR* odds ratio, *95% CI* ninety–five percent confidence interval

^a ^Crude estimate

^b ^Adjusted for gender and age (continuous)

^c ^Dichotomized based on the 50th percentile

^d ^Only working subjects (N = 3843), excluding students, pensioners, unemployed, and those on sick or parental leave

### Relation between cold exposure and incident Raynaud’s phenomenon

There was an association between leisure-time cold exposure and incident RP (adjusted OR 1.06; 95% CI 1.01–1.12), but not occupational exposure among working subjects (adjusted OR 1.01; 95% CI 0.96–1.07), after adjusting for age and gender (Table 4). Ever-occurrence of cold injury affecting the hands *at baseline* was reported by 217 women (8.1%) and 287, men (12.5%), and was associated with incident RP (adjusted OR 2.90; 95% CI 1.97–4.27) at follow-up. In addition, having sustained a cold injury to the hands *since baseline* was reported by 111 women (4.1%) and 148 men (6.5%), and was also associated with incident disease (adjusted OR 3.92; 95% CI 2.60–5.90). The annual incidence of cold injuries affecting the hands was 0.7% women and 1.1% for men. Analyzing the severity of reported cold injuries, most had only experienced superficial injuries, causing white spots on the hands. Dermal or deeper engagement (indicated by the presence of clear or hemorrhagic blistering) was reported by 1.0% of women and 6.5% of men for injuries at baseline, and 1.8% and 6.8% for injuries during follow-up, respectively. Regarding time relations, 84 women and 83 men had specified complete time series for year of first occurrence of both cold injury and RP. Cold injury had preceded RP in 40 women (47.6%) and 30 men (36.1%). The same year of onset for both conditions was reported by 37 (44.0%) and 47 (56.6%), while RP had preceded cold injury in 7 (8.3%) and 6 (7.2%), respectively. The were no significant gender differences in the time series (*p* = 0.26).

## Discussion

### Main findings

The prevalence of RP in the follow-up survey was 14.5% among women and 12.7% among men. The annual incidence proportion of RP in northern Sweden was 0.7% and 0.9%, respectively. The annual remission proportion was 4.4% for women and 5.5% for men. New-onset cold injury was associated with incident RP (OR 3.92; 95% CI 2.60–5.90), after adjusting for age and gender.

### Interpretation and comparison with other studies

The prevalence of RP in the baseline and follow-up surveys of around 12–15% was comparable with the roughly 12% that was reported in a Finnish population-based study [5]. The condition was more common among women, which is in line with previous research [1]. The median age of onset of 31 years for women and 35 years for men (as shown in Table 2) was quite similar to the results of a meta-analysis on longitudinal studies on RP (10 studies; 639 subjects), where the mean age of onset was 34 years [11]. The annual incidence of RP (0.7% in women and 0.9% in men) in our study was higher than what was described in the previous prospective study by Suter et al., reporting 0.3% in women and 0.2% in men during a mean follow-up of seven years [6]. It was also higher than the French prospective study by Carpentier et al. that reported an annual incidence of 0.2% in women and 0.3% in men after about 14 years of follow-up [7]. One explanation for the differences in incidence proportions may be the more specific case definitions in the two referenced studies. Suter et al. used a validated instrument during interviewing, required current symptoms (within 12 months), and mandated that three out of four clinical criteria were fulfilled. In comparison, our study was based on a single questionnaire item, and the diagnosis was not confirmed by interview. The study by Carpentier et al. also illustrated this effect very clearly, since the questionnaire-based cumulative proportion of RP was originally roughly 14%, but effectively reduced to merely 3.5% after careful medical evaluation by an experienced physician. The disparity in incidence proportions between studies may also be explained by differences in climate, since our study was conducted during a subarctic winter season at latitudes between 62–69°N, the study by Suter et al. during the entire year in Framingham (latitude 42°N), and the study by Carpentier et al. during all seasons in Tarentaise (latitude 46°N). This concept is further supported by the fact that out study showed a significant impact of cold exposure, where leisure-time cold exposure and cold injuries to the hands was associated with incident RP, whereas the study by Suter et al. found no effect of collecting data during the winter season. Our study indicated that men had a significantly higher incidence proportion than women, whereas no such gender differences were reported by Suter et al. and Carpentier et al. The annual remission proportion of roughly 4–6% in our study was somewhat lower than the 9% reported by Suter et al. but higher than the 3% found by Carpentier et al. There disparities may also be explained by differences in case definition and study design.

Our study was not designed to establish whether RP was of primary or secondary origin. However, the characteristics (as outlined in Table 2) suggest that most had current symptoms (during the last two years), but only of mild to moderate severity, without progression in terms of attack frequency or anatomical distribution. These features, together with the female predominance and age distribution, could be argued to support that RP was mostly of primary origin, although this cannot be established with certainty. Looking more closely at gender differences in RP characteristics, women reported earlier age of onset and less severe disease with regards to both frequency of attacks and anatomical distribution (Table 2). This could be due to a larger proportion of secondary RP among men, e.g. due to hand-arm vibration or cold injury. Further studies are needed to clarify this.

The annual incidence of cold injuries affecting the hands of 0.7% for women and 1.1% for men in our study can be compared to a Finnish population-based study, where the annual occurrence of severe frostbite was 0.6% for women and 1.1% for men [24]. There were statistically significant associations between cold injuries affecting the hands (both at baseline and during follow-up) and incident RP. Importantly, a subgroup analysis on time relations supported that cold injury often preceded or occurred in close conjunction with the onset of RP. In contrast, RP debuted before cold injury in only roughly 7–8% of cases, suggesting that this is a much rarer event. Several distinct mechanistic pathways have been suggested for the link between cold injury and vascular pathology [28, 29]. First of all, intense cold exposure induces an increased sympathetic tone, resulting in vasoconstriction. Cold injury is also associated with extracellular osmotic shifts and hemoconcentration, leading to increased blood viscosity. Local tissue injury induces formation of free radicals, which can cause endothelial damage, and lead to changes in expression of vasoactive substances, such as prostaglandins and thromboxane. Endothelial damage can also occur as a direct mechanical effect of the formation of ice crystals in the intra- and extracellular space, with loss of NO-dependent vasodilatory capacity. Finally, cold-induced autonomic nerve injury can also shift the balance towards increased vasoconstriction. Thus, there is both epidemiological and mechanistic support for the hypothesis that cold injury can be an etiological factor in the development of RP, by altering the microvascular regulation of the hands [3].

Other than cold injury, there were few of the selected baseline variables that predicted incidence or remission of RP. Age had a significant effect, with lower incidence and higher remission proportion with increasing age. This may be due to the fact that the peripheral vasoregulatory response attenuates with age, as well as behavioral changes with decreased ambient cold exposure among older subjects [4]. High body mass index had a protective effect, which has been suggested to be related to the isolating properties of subcutaneous fat [30]. In crude analyses, there was a weak association between daily snuff use and incident RP, but not daily smoking. Both tobacco products contain nicotine with vasoconstrictive properties. However, habitual snuff users can be more exposed to nicotine than smokers, which may explain the difference in effect [31]. Hypertension at baseline was associated with remission of RP, which is a novel finding. This could possibly be explained by the fact that a higher perfusion pressure in the fingers is beneficial among subjects with peripheral vasospastic tendencies [32]. High cold exposure during leisure time, but not during work, was associated with incident RP. This might be due to the fact that outdoor leisure-time activities were the dominant sources of ambient cold exposure in this cohort [4].

### Limitations

The response rate in our study was rather low and this could have affected the generalizability of results. There was an underrepresentation of younger age groups among responders, but only minor distortions regarding gender and geographical location, when compared to the original sampling frame (Additional file 1). Previously, it has been argued that large-scale population-based recruitment strategies may provide good generalizability even if the response rate is low, under the condition other that sources of sampling bias are limited [33], which is believed to be the case in our study. The switch from a postal to a digital survey may have deterred subjects that are unaccustomed to computer tasks from responding, inducing a systematic bias. However, subjects who were unable or reluctant to answer digitally were given the option to receive a paper survey. Questionnaires are generally inferior to field measurements of exposure, and clinically gathered data. On the other hand, surveys are cost-effective when covering larger cohorts, but the results need to be confirmed by studies of other design.

It is known that certain medications such as antihypertensive agents can affect the expression of RP [17]. For instance, adrenergic beta-antagonists can aggravate RP, while calcium channel blockers actually increase peripheral perfusion and can be used as a treatment for RP. Thus, the lack of detailed information on substance and dose of prescription drugs in our study is a limitation. In the same manner, although several rheumatic conditions (e.g. systemic sclerosis and systemic lupus erythematosus) are associated with RP, specific questions regarding the presence of rheumatic diseases were not included in the questionnaires. On a final note, our study was not designed to separate between primary and secondary RP, and gathering such information would likely have aided in the interpretation of the results.

### Strengths and implications

To the authors’ knowledge, this is by far the largest population-based prospective study on RP, and the first in a Scandinavian setting, where the condition is prevalent. Through detailed surveys, many relevant covariates could be investigated. The questionnaires were distributed during the same season at both baseline and follow-up, so that cold exposure would be comparable. Also, since previous studies have reported on gender differences both regarding occurrence [1] and risk factors [34], analyses were either stratified or adjusted for gender. Since this study shows that cold exposure and related injuries increase the risk of suffering from RP, preventive measures are motivated. First of all, information campaigns could be launched, targeting the general population, to spread knowledge about the risks of intense cold exposure. Secondly, highly cold-exposed occupational groups could benefit from occupational health surveillance interventions, such as technical and medical risk assessment, and periodical medical check-ups.

## Conclusions

In the general population of northern Sweden, Raynaud’s phenomenon is a common but variable condition, where symptoms may remit over time. Men had a higher incidence proportion than women. The results support a possible causal pathway where cold injury can precede the onset of Raynaud’s phenomenon.

## Supplementary Information

**Additional file 1**. Responder analysis for the baseline and follow-up surveys.

## Data Availability

The datasets generated and analyzed during the current study are not publicly available due to restrictions in ethical and data management approvals, but are available from the corresponding author on reasonable request.

## Abbreviations

RP: Raynaud’s phenomenon
CHINS: Cold and Health In Northern Sweden
IQR: Interquartile range
NRS: Numerical rating scale
BMI: Body mass index
ISCO: International Standard Classification of Occupations
OR: Odds ratio
95% CI: Ninety-five percent confidence interval
