# Supplementary material for: Incidence, remission, and persistence of Raynaud’s phenomenon in the general population of northern Sweden: a prospective study
Source: BMC Rheumatol. 2022 Jul 21;6:41. doi: 10.1186/s41927-022-00272-0 (PMC9301854; doi:10.1186/s41927-022-00272-0)
Supplement: Supplementary file 1 — Additional file 1. Responder analysis for the baseline and follow-up surveys. [file 41927_2022_272_MOESM1_ESM.pdf]

Additional file 1: Distribution of age, gender, and geographical location for the Cold and Health In Northern Sweden (CHINS) 2015 sampling frame, CHINS2015 responders, and CHINS2021 responders.

Title of paper: Incidence, remission, and persistence of Raynaud's phenomenon in the general population of northern Sweden – a prospective study

Authors: Albin Stjernbrandt, Hans Pettersson, Ronnie Lundström, Ingrid Liljelind, Tohr Nilsson, Jens Wahlström

Journal: BMC Rheumatology

		CHINS2015 sampling frame		CHINS2015 responders (baseline)		CHINS2021 responders (follow-up)	
**Variable**		N	%	N	%	N	%
**Age group (years) [a]**							
18–31		9,328	26.5	1,816	14.4	476	9.5
32–44		7,553	21.5	2,167	17.2	872	17.4
45–57		8,894	25.3	3,486	27.6	1,603	32.0
58–70		9,369	26.7	5,158	40.8	2,066	41.2
**Gender**							
Women		17,589	50.0	6,886	54.5	2,703	53.9
Men		17,555	50.0	5,741	45.5	2,314	46.1
**County**							
Norrbotten		9,036	25.7	3,115	24.7	1,179	23.5
Västerbotten		10,627	30.2	3,944	31.2	1,710	34.1
Västernorrland		8,905	25.3	3,203	25.4	1,202	24.0
Jämtland		6,576	18.7	2,365	18.7	926	18.5
**Gender**	**Age group (years) [a]**						
Women	18–31	4,651	26.4	1,043	15.1	277	10.2
	32–44	3,806	21.6	1,275	18.5	530	19.6
	45–57	4,480	25.5	1,943	28.2	868	32.1
	58–70	4,652	26.4	2,625	38.1	1,028	38.0
Men	18–31	4,677	26.6	773	13.5	199	8.6
	32–44	3,747	21.3	892	15.5	342	14.8
	45–57	4,414	25.1	1,543	26.9	735	31.8
	58–70	4,717	26.9	2,533	44.1	1,038	44.9
**Gender**	**County**						
Women	Norrbotten	4,542	25.8	1,679	24.4	617	22.8
	Västerbotten	5,268	30.0	2,120	30.8	920	34.0
	Västernorrland	4,490	25.5	1,778	25.8	660	24.4
	Jämtland	3,289	18.7	1,309	19.0	506	18.7
Men	Norrbotten	4,494	25.6	1,436	25.0	562	24.3
	Västerbotten	5,359	30.5	1,824	31.8	790	34.1
	Västernorrland	4,415	25.1	1,425	24.8	542	23.4
	Jämtland	3,287	18.7	1,056	18.4	420	18.2

[a] Age at enrollment (2015).
